# The impact of stigma on mental health and quality of life of infertile women: A systematic review

**DOI:** 10.3389/fpsyg.2022.1093459

**Published:** 2023-01-09

**Authors:** Yue Xie, Yue Ren, Changmin Niu, Ying Zheng, Ping Yu, Lin Li

**Affiliations:** ^1^School of Nursing and Public Health, Yangzhou University, Yangzhou, China; ^2^Affiliated Hospital of Yangzhou University, Yangzhou, China

**Keywords:** mental health, infertility, stigma, quality of life, review

## Abstract

**Introduction:**

The stigma of not giving birth to children affects approximately 53. 08~64% of female infertility patients worldwide. This stigma not only causes harm to the mental health of these infertility patients, but also affects their quality of life, making them bear the adverse social consequences such as domestic violence, marriage breakdown, or even delay in receiving the treatment. Therefore, it is crucial to have a deep understanding of the patients' stigma and effective intervention in alleviating it.

**Aims/Question:**

This study aims to discuss and summarize the stigma in infertile women and its impact on patients, and to provide a theoretical basis for the clinical treatment and nursing intervention of disease stigma in infertile female patients.

**Methods:**

The literature search used four English databases (Cochrane Library, EMBASE, Web of Science, and PubMed) and two Chinese databases (CNKI and Wanfang). The search time of the literature ranges from the establishment of the library to 2022, with no language restriction.

**Results:**

The review included 28 studies, with 20 cross-sectional studies and 8 qualitative studies. This study found that social support, living environment, education level, occupation, and fertility awareness were the major influencing factors of infertility stigma.

**Conclusions:**

Infertility stigma can bring heavy mental pressure and psychological burden to female infertility patients and affect their quality of life. Therefore, effective and targeted psychological interventions should be developed to reduce the patients' stigma and improve their quality of life.

**Implications for practice:**

Healthcare workers must develop targeted nursing interventions, provide professional counseling services to reduce the level of stigma in female infertility patients, alleviate fertility stress, and improve their quality of life.

## 1. Introduction

Infertility refers to a situation in which both the husband and the wife want to have children, have a normal sexual life, and have not used contraception for more than a year but still cannot conceive (Zegers-Hochschild et al., 2017). Due to the influence of adverse factors such as environmental pollution, work pressure, and changes in living habits, the number of infertility patients increases annually (Fu et al., 2015).

Infertility has become a major public health concern worldwide (Tsevat et al., 2017). According to the WHO statistics, infertility has become the third major disease in the twenty-first century, after tumors, cardiovascular, and cerebrovascular diseases, threatening human health (Mascarenhas et al., 2013). Studies have shown that the proportion of infertile women is between 3.5 and 16.7% in developed countries and between 6.9 and 9.3% in developing countries. About 72~80 million women of childbearing age worldwide currently have infertility (Khakbazan et al., 2020).

Although the male-female ratio of infertility patients has increased recently, the female infertility patients seem to be more psychologically stressed and bear the brunt of more severe social consequences in the face of infertility. Studies have revealed that both seeking and not seeking treatments can lead to an emotional distress in infertile women (McQuillan et al., 2003). In traditional cultural societies, not having children is often considered a woman's fault (Tiu et al., 2018). Infertile women are often observed as worthless or alien (Fledderjohann, 2012; Zhang et al., 2021). Some unreasonable social cognition and prejudice make many infertile women suffer different forms of discrimination in their lives and bear the social consequences such as violence from family, marriage breakdown, or malicious evaluation from surrounding people (Batool and de Visser, 2014; Dag et al., 2014; Kaya and Oskay, 2020). In Turkey, childless women are observed as “trees of no fruit” (Koçyigit, 2012). A similar phenomenon has occurred in Jordan, where infertile women are described as “The wings are broken,” “dead wood,” and “half male and half female” (Daibes et al., 2018). In this social environment, female infertility patients are highly susceptible to the stigmatization of the disease (Yilmaz and Kavak, 2019). The concept of stigma was first introduced by the American sociologist (Goffman, 2009), defining it as “an indecent social mark.” When some people are given this label, they are often treated with contempt by ordinary people (Goffman, 1963). It can also lead to humiliation and discrimination for infertile women suffering from identity stigma because they believe they cannot meet social expectations, resulting in painful conditions such as anxiety and depression (Davern and O'Donnell, 2018). Recently, it has been studied primarily in patients with cancer or stroke (Fujisawa and Hagiwara, 2015; Zhu et al., 2019). However, there is no uniform definition of stigma in infertility patients. Rie Yokota, a Japan scholar, stated that after being diagnosed with infertility, the female infertility patients suffer from feelings of guilt, shame, and self-depreciation due to fear of rejection and social and family humiliation, which lead to negative emotions, rigid marital relationships, and decreased quality of life, affecting the normal life of patients (Yokota et al., 2022).

The stigma of the disease negatively affects the female infertility patient's body and mind (Ying et al., 2015; Freeman et al., 2018). In addition, this stigma can severely affect the patient's social skills, segregating the patient from some positive social interactions (Cousineau and Domar, 2007; Slade et al., 2007). Due to the stigma of the disease, some patients are even unwilling to contact the members of the society at large, shut themselves up, produce more severe anxiety and depression-related symptoms, or delay medical treatment, thereby aggravating or impeding infertility treatment (Miles et al., 2009; Schwerdtfeger and Shreffler, 2009; Öztürk R. et al., 2021).

The stigmatization of the disease has such a tremendous negative impact on the patient's body and mind, and the quality of daily life also declines. This review aims to investigate whether disease stigma affects the mental health and the quality of life of women experiencing fertility difficulties and provide a reasonable method for the medical staff to develop effective interventions in the future, reduce patient stigma, reduce the patient psychological burden, and improve the quality of life.

## 2. Review

### 2.1. Objective

This retrospective article aims to summarize the impact of stigma on infertile women's mental health and quality of life to provide supporting evidence. Possible countermeasures were explored to develop appropriate interventions for patients in the future.

### 2.2. Methods

#### 2.2.1. Design

This systematic review selects articles for inclusion based on the System Review and Meta-Analysis Preferred Reporting Project (PRISMA) guidelines (Page et al., 2021; Supplementary material). The Strengthening the Reporting of Observational Studies in Epidemiology guidelines (Von Elm et al., 2007) were used to assess the quality of articles. The quality of all qualitative studies was assessed using Evidence-Based Care Guidelines tool.

#### 2.2.1. Literature search

We conducted a comprehensive literature search without language restrictions using the databases of the Cochrane Library, EMBASE, Web of Science, PubMed, Wanfang data, and CNKI from their establishment until July 2022. Computer searches used Medical Subject Headings and keywords, including “infertility,” “subfertility,” “barrenness,” “sterility,” “reproductive sterility,” “stigma,” “social stigma,” “perceived stigma,” “shame,” “discriminate,” “psychological,” “psychological stress,” “mental health,” and “quality of life.” Preliminary screening is carried out by the thesis title and abstract. In addition, relevant citations from the included studies were searched by hand.

### 2.3. Criteria for inclusion and exclusion in the literature

**Inclusion criteria:** (a) These studies must have been published, and the research methodology should be a cross-sectional study associated with female infertility stigma; (b) the subjects were infertile women; (d) the outcome measures included the association between stigma and mental health, or between stigma and quality of life. (c) There is no restriction on the language and year of publication of the literature.

**Exclusion criteria:** (a) Literature that does not match the content of this article. (b) The literature data are incomplete, and complete information cannot be obtained after contacting the author. (c) Meta, systematic reviews, and reviews of the literature. (d) The quality evaluation is low in the literature.

### 2.4. Extraction of data

Two researchers independently conducted literature screening, data extraction, and cross-checking. If there was any disagreement, it was passed after resolving through a third-party negotiation. First, the researchers screened the literature by reading the title of the paper in order to exclude literature that was obviously not relevant to the study. They then read the abstract and full text to determine whether the study was included or not. The extracted content included: title, author, publication time, country of publication, research type, research content, and research results.

### 2.5. Quality appraisal

The quality of articles was evaluated independently by two reviewers using Strengthening the Reporting of Observational Studies in Epidemiology guidelines (STROBE) (University of Bern, 2009). In case of disagreement, a third researcher was invited to participate in the discussion to reach a consensus. This guide has 22 items with a total score of 22 points. Each item received one point if it met the criteria. If the description was insufficient or nonexistent, it received a score of zero. A score of ≥17 counts as a high-quality article. A score between 11 and 16 counts as a medium-quality article. A score of ≥ 10 counts as a low-quality article.

The 20 studies included in this study were assessed for quality, and so no low-quality articles were found. Concurrently, 10 items are high-quality articles. In addition, there are 10 out of 20 medium-quality articles. The details can be found in Table 1.

**Table 1:** Variables assessed and methodological quality of the studies included in the review.

**References**	**Mental health**	**Quality of life**	**The quality of the evidence**	**Total**
Dyer et al. (2002)	■		High	8
Slade et al. (2007)	■		High	18
Donkor and Sandall (2007)	■		Moderate	15
Nieuwenhuis et al. (2009)	■		High	7
Galhardo et al. (2011)	■		Moderate	15
Nahar and Richters (2011)	■		High	9
Fledderjohann (2012)	■		High	9
Galhardo et al. (2013)	■		Moderate	15
Tabong and Adongo (2013)	■		High	8
Galhardo et al. (2014)	■		High	18
Li et al. (2017)	■		High	19
Daibes et al. (2018)	■		High	9
Yilmaz and Kavak (2019)	■		Moderate	15
Roberts et al. (2020)	■		High	17
Kaya and Oskay (2020)	■		High	17
Van Rooij et al. (2020)		■	Moderate	16
Ofosu-Budu and Hanninen (2020)	■		High	9
Ozturk A. et al. (2021)	■		High	17
Öztürk R. et al. (2021)	■		Moderate	16
Lin et al. (2021)	■		Moderate	15
Fang et al. (2021)	■		Moderate	14
Jing et al. (2021)		■	High	18
Zhang et al. (2021)	■		Moderate	15
Taebi et al. (2021)	■		High	9
Küçükkaya and Kiliç (2022)	■		Moderate	15
Yokota et al. (2022)	■		High	18
Jing et al. (2022)		■	High	17
Zhao et al. (2022)	■		High	17

Note: ■ represents the outcome variables of the study.

The reviewers assessed the quality of these eight qualitative studies using evidence-based care guidelines (Russell and Gregory, 2003). Scoring scales ≥ 5 are classified as high-quality studies, and scoring scales ≤ 4 are classified as low-quality studies. After evaluation, 8 out of 8 studies were identified as high-quality studies. The scores are given in Table 1.

In qualitative studies, the research questions are accurate and true, the methods adopted are consistent with the purpose of the research, and the collected data were complete. The phenomena studied can be clearly described, and the results obtained were logical. However, one study did not mention the previous fertility of residents (Nieuwenhuis et al., 2009).

## 3. Results

### 3.1. Characteristics of the included literature

**Figure 1:**
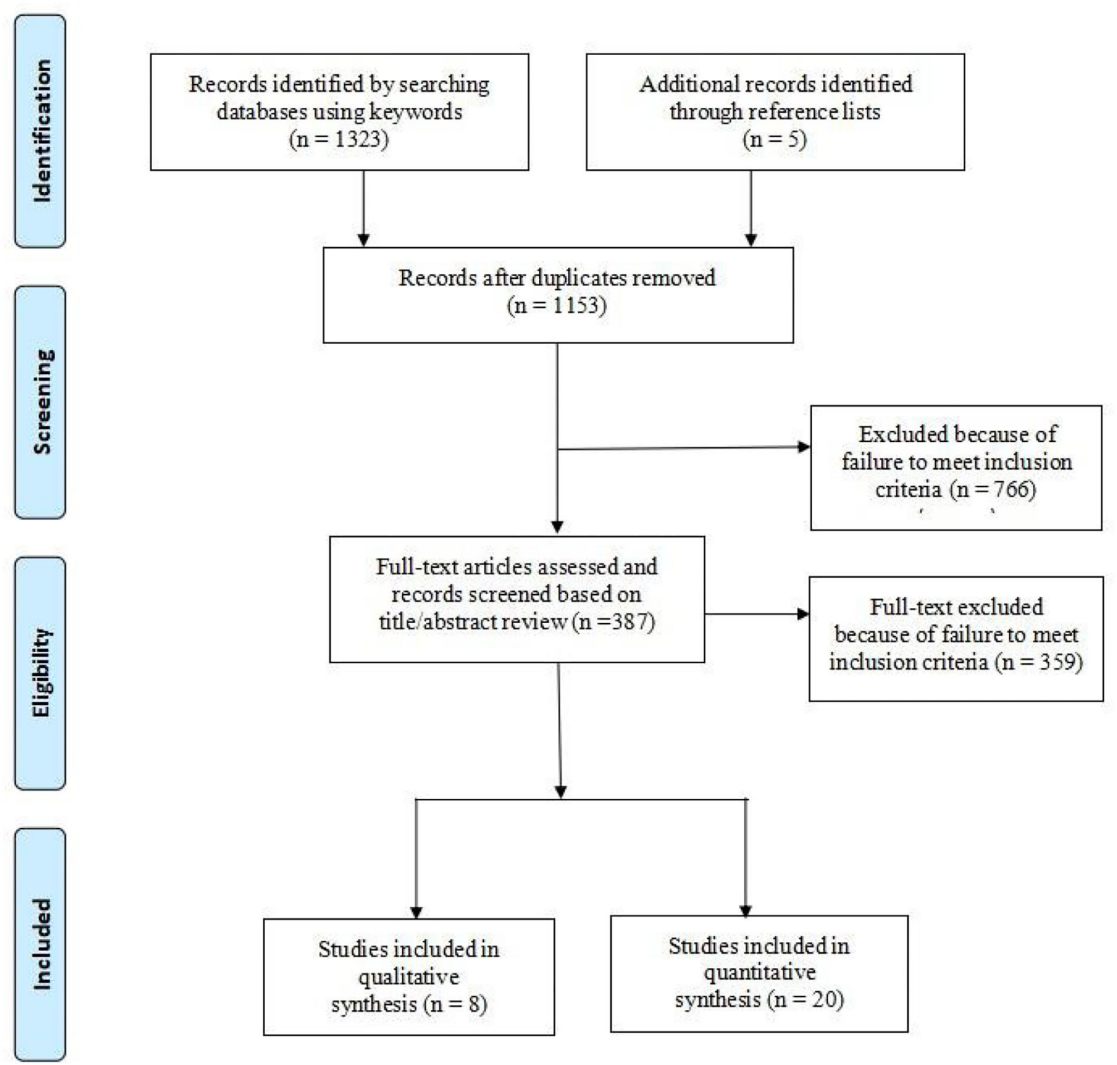
PRISMA flow diagram.

This review includes 28 studies, with 20 cross-sectional and 8 qualitative studies (Figure 1). All studies were published in peer-reviewed journals. The article included different populations from 13 countries with 8,193 participants. The four studies were conducted in Europe (Portugal, United Kingdom), eight in the Middle East (Iran, Turkey, and Jordan), ten in East Asia (China and Japan), five in Africa (Ghana, Nigeria, and South Africa), and three in North America (United States of America).

This paper includes 28 studies, mainly discussing the current disease stigma in infertile women, the impact of stigma on patients' mental health and quality of life, and clinical interventions for stigma. In the cross-sectional studies, 17 studies focused on the psychological condition of infertile women and 3 on the quality of life of infertile women. All qualitative studies focused on the psychological problems of infertile patients, and two also focused on patients' quality of life.

The results of the review are presented in three tables: the effects of the disease stigma on the mental health of females with infertility (Table 2), quality of life (Table 3), and a summary of the qualitative studies (Table 4).

**Table 2:** Summary of the effects of disease stigma on mental health in female patients with infertility.

**References**	**Design**	**Sample size**	**Instruments**	**Important findings**
Slade et al. (2007) (UK)	Cross-sectional	87 Women with infertility, 64 men with infertility	SCQ, FPI, HADS	Infertile women perceive a higher sense of stigma compared to men. Stigma is directly associated with general suffering.
Donkor and Sandall (2007) (Ghana)	Cross-sectional	615 infertile women	PSS, FPI	Higher levels of perceived stigma were associated with increased infertility-related stress.
Galhardo et al. (2011) (Portugal)	Cross-sectional	100 fertile group (FG) couples; 100 infertile group (IG) couples; 100 Adoption group (AG) couples	OAS, ESS, BDI, STAI-Y	In infertile couples, wives tend to show higher levels of depression, self-judgment, and external shame can play a predictive role.
Galhardo et al. (2013) (Portugal)	Cross-sectional	309 patients (162 women and 147 men)	OAS, ESS, BDI	External stigma can be a direct predictor of infertility-related stress.
Galhardo et al. (2014) (Portugal)	Cross-sectional	162 infertile women	ESS, FPI, BDI	Stress and depression caused by external shame are directly linked.
Li et al. (2017) (China)	Cross-sectional	211 infertile women	Disease-related information questionnaire, SDS	Infertile women in China will have a sense of shame because of discrimination from the outside world, resulting in depression.
Yilmaz and Kavak (2019) (Turkey)	Cross-sectional	121 infertile women	ISS, BDI	A significantly positive correlation was found between stigma and depression as the level of stigma increased, depression increased.
Roberts et al. (2020) (USA)	Cross-sectional	74 infertile women	ISS, HSCL-10	The stigma of infertility can cause patients to develop negative emotions, such as anxiety and depression.
Kaya and Oskay (2020) (Turkey)	Cross-sectional	278 infertile women	ISS, BHS	The mental health of infertility can be affected by stigma.
Ozturk A. et al. (2021) (Turkey)	Cross-sectional	298 infertile women	ISS, BDI	Depression increased as stigmatization increased.
Öztürk R. et al. (2021) (USA)	Cross-sectional	786 women	PSS	Women were subjected to emotional and family violence because of infertility, and there were a huge psychological burden and a sense of stigma.
Fang et al. (2021) (China)	Cross-sectional	369 couples with fertility difficulties	The self-designed stigma scale; K10	Psychological distress in infertile patients increases with the increase in stigma.
Zhang et al. (2021) (China)	Cross-sectional	254 infertile women	ISS	In Zhejiang, China, infertile women experience moderate to high levels of stigma, which can lead to psychological stress, mainly in the form of social withdrawal.
Lin et al. (2021) (China)	Cross-sectional	245 infertile women	A 7-item Infertility Stigma Scale; POMS	Self-stigma can lead to negative psychological.
Küçükkaya and Kiliç (2022) (Turkey)	Cross-sectional	198 infertile women	ISS, IDS	The psychological effect of infertility increased among women as the Infertility Stigma Scale total scores and its sub-dimension scores increased.
Yokota et al. (2022) (JPN)	Cross-sectional	254 infertile women	ISS, HADS	Stigma can predict anxiety, depression, and psychological distress in patients.
Zhao et al. (2022) (China)	Cross-sectional	266 infertile women	ISS,SADS	The stigma can cause patients to have social avoidance tendencies and distress in actual interactions.

SCQ, Social Communication Questionnaire; FPI, Fertility Problem Inventory; BAI, Beck Anxiety Inventory; HADS, Hospital Anxiety and Depression Scale; ESS, Experience of Shame Scale; BDI, Beck Depression Inventory; SDS, Self-Rating Depression Scale; PSS, Perceived Stress Scale; ISS, Infertility Stigma Scale; BHS, Beck Hopelessness Scale; STAI-Y, State-Trait Anxiety Inventory; HSCL-10, The Hopkins Symptoms Checklist-10; POMS, Based on the Profile of Mood States; IDS, The Infertility Distress Scale; SADS, Social Avoidance and Distress Scale; OAS, Others as Shamer Scale; K10, The 10-item Kessler Psychological Distress Scale.

**Table 3:** Summary of the effects of disease stigma on the quality of daily life in female patients with infertility.

**References**	**Design**	**Sample size**	**Instruments**	**Findings**
Van Rooij et al. (2020) (Ghana)	Cross-sectional	38 women, 11 men	ISS, FertiQoL	Stigmatization was negatively correlated with fertility quality of life.
Jing et al. (2021) (China)	Cross-sectional	768 infertile women	ISS, FertiQoL	The stigma affects a patient's quality of life.
Jing et al. (2022) (China)	Cross-sectional	588 infertile women	ISS, FertiQoL	The higher the stigma of women receiving treatment, the worse the quality of life.

ISS, Infertility Stigma Scale; FertiQoL, Fertility quality of life.

**Table 4:** Summary of the qualitative studies.

**References**	**Design**	**Sample size**	**Instruments**	**Findings**
Dyer et al. (2002) (South Africa)	Qualitative	30 infertile women	In-depth interviews	Many women described how others cursed them. They suffer great personal suffering within themselves and can have serious social consequences.
Nieuwenhuis et al. (2009) (Nigeria)	Qualitative	7 infertile men, 8 infertile women	In-depth interviews	If a woman fails to conceive, she may be taunted by her in-laws, neighbors, and relatives, which can have severe social, psychological, and economic impacts on her life.
Nahar and Richters (2011) (UK)	Qualitative	31 infertile women	The life-history method	In rural areas, women unable to have children are strongly humiliated and belittled. They feel socially isolated and abandoned by their families.
Fledderjohann (2012) (USA)	Qualitative	107 infertile women	Semi-structured interviews	Infertile women face severe social stigma. Many women believe that they bear a higher responsibility for infertility than men, which puts them under greater psychological stress and faces more severe social consequences.
Tabong and Adongo (2013) (Ghana)	Qualitative	15 childless couples, 45 couples with children	In-depth interviews	Couples unable to have children are ostracized by society and criticized by neighbors, resulting in the physical and mental impact on infertile patients.
Daibes et al. (2018) (Jordan)	Qualitative	14 infertile women	Semi-structured interviews	Infertile women reported that they were stigmatized by society and the ego due to infertility, manifested in social exclusion and isolation, which led to increased psychological stress and a decline in their quality of life.
Ofosu-Budu and Hanninen (2020) (Ghana)	Qualitative	30 infertile women	phenomenological methods	Some women revealed that, due to the high stigma, they are considering leaving their homes to reduce discrimination and exclusion from the outside world.
Taebi et al. (2021) (Iran)	Qualitative	17 infertile women	Semi-structured interviews	Study participants were often badly evaluated by the outside world and internalized the stigma as worthless, adding to their own guilt.

### 3.2. Stigma in female patients with infertility

Studies have revealed that infertile women worldwide are stigmatized for infertility (Whiteford and Gonzalez, 1995; Jansen and Saint Onge, 2015; Anokye et al., 2017; Zhang et al., 2021). Although infertile women in different countries and regions may differ in stigma due to cultural, religious, ideological, economic, and other differences (Karaca and Unsal, 2015), there is no doubt that all infertile women with stigma have poor mental health and quality of daily life (Brown, 2022; Yokota et al., 2022).

Social support is essential and is significantly associated with stress levels in infertile women (Gibson, 2000; Martins et al., 2011). Family and partners are important for infertile women to seek outside help. However, the social stigma and personal shame of infertility force infertile women to conceal their condition, preferring to keep their infertility a secret rather than communicate with family or friends. They can use self-isolation to reduce the effects and injuries caused by the surrounding environment (Whiteford and Gonzalez, 1995; Ramazan et al., 2009; Karaca and Unsal, 2015). In a study of 21 infertile women, the patients highlighted their difficulties with friends who had children and found that they experienced social withdrawal at family and friend gatherings because they felt marginalized (Pedro, 2015). In another study, the authors found that infertile women deliberately avoided topics related to family or children to avoid sad or uncomfortable conversations (Remennick, 2000). These negative social interactions may reduce infertile women's perception of social support, further increase patients' stigma, threaten their mental health, and exacerbate anxiety and depression.

In addition to social support, living environment, education level, occupation, and fertility awareness can also impact patients' stigma. The living environment in which infertile women live can impact their stigma. Compared to developed countries, women who live in traditional societies or accept traditional cultural beliefs believe that infertility places the most significant burden, stigma, and suffering (Greil et al., 2010; Ying et al., 2015). This may be because, in countries with sociocultural and traditional notions, the status of being a mother is important for a woman, and failure to take on this responsibility can be socially ostracized and humiliated (Jansen and Saint Onge, 2015). This makes many female infertility patients more eager to get pregnant and more sensitive to words such as “pregnancy” and “child,” which increase their fertility pressure (Zhang et al., 2021). In addition to sociocultural factors in different countries leading to female stigma, different regions in each country produce different levels of stigma. Studies have indicated that infertile women with rural lifestyles are more discriminated against than infertile women with urban lifestyles (Donkor and Sandall, 2007; Li et al., 2017; Yilmaz and Kavak, 2019; Ozturk A. et al., 2021). Traditional culture is often more prevalent in rural areas, and because of the rapid spread of information, infertile women are more vulnerable to social isolation and neglect (Koçyigit, 2012). The family environment can also increase the stigma among infertile women. Two studies have found that living with parents may increase women's anxiety and make them more pressurized to have children (Li et al., 2017; Yokota et al., 2022).

Some studies have found that stigma is associated with educational level (Donkor and Sandall, 2007; Lin et al., 2021; Ozturk A. et al., 2021; Zhang et al., 2021; Küçükkaya and Kiliç, 2022; Zhao et al., 2022). Patients with higher education levels have lower stigma than those with lower education levels, and patients with lower education levels are more likely to fall into an inferiority complex, leading to more severe stigma (Donkor and Sandall, 2007; Alhassan et al., 2014). This may be because highly educated women have more opportunities and abilities to learn about diseases. This helps them have access to real-time, effective treatment and the ability to overcome discrimination and infertility-related stigma in a positive manner (Alhassan et al., 2014; Jing et al., 2021).

Occupation is also an important factor in the perception of stigma (Donkor and Sandall, 2007; Lin et al., 2021; Ozturk A. et al., 2021; Zhang et al., 2021; Küçükkaya and Kiliç, 2022; Zhao et al., 2022). On the one hand, occupation may be related to income level. One study observed that the psychological distress of infertile women whose income was lower than their expenditure was more severe (Küçükkaya and Kiliç, 2022). Other studies have also shown that the cost of treatment can carry tremendous psychological stress on infertile patients (McQuillan et al., 2003; Ozkan and Baysal, 2006). Conversely, women with higher incomes experience a smaller psychological burden. They can devote their energy to work and career, distract themselves from the disease, and at the same time, learn some psychological skills to strengthen their psychological strength (Zhang et al., 2021). On the other hand, it may be related to whether the infertile woman is working or not. Women with fixed jobs and incomes can earn a sense of accomplishment through work and also have a stable source of income, ensuring their daily quality of life, and their guilt and sensitivity to diseases will be relatively low compared to unemployed women (Alhassan et al., 2014; Jing et al., 2021). Homemakers cannot obtain information about infertility and its consequences because they have little communication with the outside world. The work creates an environment for infertile women to help cope with infertility and support women, so that they can express their ideas and discuss and communicate with colleagues, relieving pressure on infertile patients to a certain extent (Nahar and van der Geest, 2014; Verma and Baniya, 2016).

An Iranian study revealed that the higher the level of irrational reproductive cognition, the higher the depression (Farzadi and Ghasemzadeh, 2008). This may be because infertile patients with a high level of irrational fertility cognition will have a strong willingness to have children, and when the willingness to have children cannot be realized, the patient will think that she is not a complete woman, resulting in self-deprecating cognition, triggering the sense of stigma (Fekkes et al., 2003; Farzadi and Ghasemzadeh, 2008).

### 3.3. Effects of the stigma on mental health female in infertile women

Because of fertility defects, infertile women suffer a double whammy from themselves and the outside world (Keramat et al., 2013; Yeshua-Katz, 2018). First, it makes patients very prone to stigma, and the stigma increases the psychological burden of patients, causing them to fall into negative emotions such as pain, inferiority, anxiety, depression, etc., which seriously affects the patient's physical and mental health and the treatment of diseases (Yagmur and Oltuluoglu, 2012; Li et al., 2017; Yilmaz and Kavak, 2019). Infertility patients from different cultural backgrounds are constantly faced with various unavoidable problems, which can cause significant psychological damage (Slade et al., 2007; Kaya and Oskay, 2020; Yokota et al., 2022). In this review, 17 out of 28 studies explored the impact of stigma on infertile women's mental health. Among these, 15 out of 28 studies found that stigma was significantly positively correlated with anxiety and depression, and the negative psychological emotions of infertile patients would increase with the stigma caused by infertility (Li et al., 2017). The remaining two studies focused on patients' feelings of hopelessness and sadness. One study of infertile women in Turkey found that female infertile patients felt hopelessness, which increased with the stigma (Kaya and Oskay, 2020). Another study from China compared their data with those of other infertility studies in China and found that women who had difficulty getting pregnant reported higher levels of sadness than healthy women (Zhao et al., 2022).

According to the literature, some couples view infertility as the enemy of life (Yari et al., 2019; Alamin et al., 2020). Furthermore, a study found that after 12 months of treatment, the suffering of both unpregnant men and women in the social sphere increased (Schmidt et al., 2005). In this review, three studies compared infertility couples and found that women scored higher on stress, anxiety, and depression than men (Slade et al., 2007; Kaya and Oskay, 2020; Fang et al., 2021). In other words, women face a higher psychological stress in the face of infertility and are more likely to suffer stigma because of fertility difficulties. This could be because cultural factors and gender roles make women more likely than men to feel ashamed and negatively self-critical (Galhardo et al., 2011; Kaya and Oskay, 2020).

In addition to directly causing the psychological distress associated with infertility, stigma can lead to reduced social support for infertility patients, further increasing their distress. Specifically, when infertile patients feel more ashamed, they seek less social support and tend to avoid social activities in the form of avoidance and marginalization, followed by greater distress (Slade et al., 2007; Ozturk A. et al., 2021; Zhang et al., 2021; Zhao et al., 2022).

### 3.4. Effects of the stigma on quality of life in infertile women

Three studies (Van Rooij et al., 2020; Jing et al., 2021, 2022) found that stigma can prevent infertile women from maintaining a normal quality of life. Society and the public are prone to give birth to a negative evaluation of infertility patients, leading to their social rejection, which increases patients' anxiety, depression, feelings of inferiority, and serious self-devaluation (Fu et al., 2015; Daibes et al., 2018). It can also lead to social withdrawal and reluctance to contact and communicate with others (Fu et al., 2015; Daibes et al., 2018). In the long run, the resulting stigma can negatively affect the quality of life of infertile women by reducing their self-esteem and self-efficacy (Remennick, 2000; Çapik et al., 2019).

Therefore, targeted, long-term interventions are needed to reduce the stigma in infertile patients to improve their quality of life. Healthcare workers should strengthen psychological counseling for infertile patients and encourage them to participate in fun group activities to improve their well-being (Domar, 2018). In addition, studies have shown that health insurance is vital for infertile women, and the purchase of insurance can reduce the burden of treatment on patients, so that their quality of life remains unchanged (Jing et al., 2022). In the future, more assistance will be required for uninsured infertile women to ensure their basic living standards.

### 3.5. Findings from the qualitative studies

Eight out of 28 qualitative studies (Dyer et al., 2002; Nieuwenhuis et al., 2009; Nahar and Richters, 2011; Fledderjohann, 2012; Tabong and Adongo, 2013; Daibes et al., 2018; Ofosu-Budu and Hanninen, 2020; Taebi et al., 2021) have found that stigma can impact a woman's daily life and psychology. These studies have reflected that infertile women face severe social stigma. Many women believe they are more responsible for infertility than men, putting themselves under more psychological stress and serious social consequences.

## 4. Intervention of disease stigma

Infertile women have different levels of stigma, either from themselves or from family, friends, classmates, and society (Slade et al., 2007). Stigma not only leads to negative emotions such as anxiety, depression, and low self-esteem in infertile patients but also leads to decreased life satisfaction, social isolation, and social avoidance, which severely affects their daily work and communication ability with others (Naab and Kwashie, 2018; Hassan et al., 2020). Therefore, there is a need for stigma interventions for infertile women. First, giving patients adequate social support. The most common source of stigma is external rejection and humiliation. A Nigerian study found that lack of support from the partner increased depression and anxiety in infertile women (Slade et al., 2007). Another study found that the adverse effects of infertility decreased significantly as infertile women received more social support (Zegers-Hochschild et al., 2017). Therefore, family members should be encouraged to give patients more companionship and care, comfort them when depressed, and prevent them from feeling alienated. Second, providing them with professional psychological counseling. When patients seek outside help, consultants must deeply understand their emotional and psychological change, understand their social avoidance and the cause of the pain, provide them with scientific cognitive intervention therapy and psychological guidance, help improve their sense of shame and sadness, and help them better fit into society and participate in regular social activities with a positive attitude. Third, improving the medical and public health service system. The high cost of infertility treatment places a huge economic burden on patients who are already reeling under great psychological stress. Developing targeted health insurance schemes or reimbursement of costs can help meet the health protection needs of patients, thereby reducing their medical burden and enabling them to receive treatment in a more positive mindset. Fourth, education is the most powerful weapon against social stigma. Infertility education should be strengthened to raise awareness of social stigma among infertile women. Simultaneously, rational fertility concepts can be introduced to patients, and their notion of fertility can be changed through active peer guidance.

## 5. Discussion

Infertility is not a simple condition. It affects the physical and mental health of patients in many ways. This review summarized 28 studies and found that infertility-related stigma can negatively impact the psychology and daily life of women with infertility. The stigma can reflect a patient's psychological attitude toward infertility and the quality of life. Patients with high stigma have high fertility pressure and are prone to anxiety and depression, affecting their quality of life.

This review found that negative social interactions or inadequate social support can increase stigma and make patients suffer more (Slade et al., 2007; Akizuki and Kai, 2008). During interpersonal communications, infertile patients with high stigma often suffer from feelings of inferiority, loneliness, and self-blame due to the fear of being ostracized by society and hurt by gossip, and avoid social activities in the form of self-seclusion, marginalization, and reluctance to communicate with family and friends, and then suffer from more severe psychological problems (Van Rooij et al., 2020; Zhang et al., 2021). A Chinese study showed that social support could protect infertile women in China from depression (Zhang et al., 2021). Stigmatized infertile women may be psychologically burdened by confiding in their husbands or other family members (Li et al., 2017). Another study discovered that infertile women were significantly less negatively affected by infertility as their social support increased (Zegers-Hochschild et al., 2017). Therefore, adequate social support may eliminate the anxiety and stress that comes with infertility. Healthcare professionals can create a comfortable environment for patients and provide appropriate psychological counseling to let them know that they are not alone and to help them face the disease with an optimistic attitude (Zhao et al., 2022).

At the same time, the study also found that improving education level and increasing employment opportunities are very important external factors in reducing the stigma of infertile women. Most articles report that the improvement in women's education level can help reduce female stigma, which may be because the improvement in women's education level will affect women's sense of identity with traditional gender concepts, and women with a higher education level have better self-regulation ability in the face of stressful events. The improvement in women's education level contributes to a better economic position and provides them with more opportunities and income in the labor market.

In traditional societies, childbirth and procreation are determinants of social status in society and the family and are seen as the main tasks of women, and women who fail to perform this responsibility are blamed and ostracized (Keskin and Babacan Güm, 2014). As a result, infertility patients are more concerned about childbearing's importance and potential significance and show a stronger sense of stigma. This review found that adverse effects on mental health only occur when infertile people agree with others to endorse the stigma and internalize this stigma as self-stigma (Corrigan and Rao, 2012). For example, women with a high level of irrational fertility in some traditional cultural environments tend to identify with the importance of childbearing. When they discover they are infertile, they define themselves as having negative traits, believing they are inferior, worthless, and flawed, and feel self-blame and guilt because they are unable to meet social norms and family expectations (Ergin et al., 2018; Huang et al., 2019). A previous study showed that individuals with lower self-esteem experienced more severe psychological distress when they experienced major stress or setbacks. Conversely, high self-esteem protects individuals from emotional distress (Fang et al., 2021). Therefore, self-esteem is an essential element that mental health professionals must consider when dealing with mental health issues in infertile patients. Research suggests that psychological resilience plays a vital role in fighting the pain and impaired quality of life specific to infertility and can be seen as a nonspecific protective factor (Herrmann et al., 2011). Therefore, when counseling patients with involuntary infertility, due consideration should be given to help improve their psychological resilience so that they learn to appreciate and accept themselves, boost their self-confidence and self-esteem, and help them cope with daily social activities with a more powerful mindset.

Based on the assessment results, regardless of the cause, women today suffer more self- and social stigma from infertility. Therefore, in the future, countries should expand the learning opportunities for girls and women, especially those living in poor areas and hailing from poor families. And to build a harmonious and inclusive culture, it is imperative to further integrate gender equality awareness into the whole process of education and even every aspect of economic and social life, and create a more gender-friendly educational and social environment. At the medical level, infertility-related stigma should be included within the scope of nursing evaluation, and the psychological state and quality of life of patients should be evaluated further. Psychosocial support is essential for infertility patients, and professionals can use psychological methods such as cognitive therapy and mindfulness therapy to improve their irrational fertility cognition level, thereby reducing the sense of shame. Meanwhile, nonmental health professionals can offer different types of stress-relieving measures such as relaxation, meditation, yoga, and other classes to help relieve negative emotions and improve the quality of life.

## 6. Limitations

This study aimed to review the effects of stigma on the psychology and quality of life of women with fertility difficulties. We recommend that medical staff and patients' families provide these hapless women adequate care and support. Concurrently, psychological intervention and public education are needed to change the patients' cognition, reduce patients' irrational cognition, and help patients respond positively to negative experiences. Although the criteria and literature quality have been strictly controlled, this paper yet has some limitations. First, the findings might get impacted because each study had a different purpose and was measured using different research tools. Second, the included studies were predominantly cross-sectional with mixed literature quality, which may impact findings. Third, this paper only includes Chinese and English literature and lacks an evaluation of non-Chinese and non-English literature.

## 7. Conclusion

Disease stigma is common in women with infertility. Being unable to have children puts a lot of stress on infertile women. And the outside world and their own shame can also increase the pain of infertile women. It seriously affects the patient's family and psychology, interferes with the mental health of the patient and her family members, and reduces the quality of their life. Therefore, the society as a whole should take into account the impact on patients such as the level of education, occupation and cost during treatment, pay attention to women's education and economic issues, and improve their social status and employment opportunities; Medical personnel should pay adequate attention to the psychological and emotional changes of infertile women and take reasonable measures to reduce the stigma of their disease and thus improve the quality of their daily life.

## 8. Relevance to clinical practice

This review has found that stigma can adversely affect infertile women's psychology and quality of life. Health workers and mental health professionals should be aware of the negative impact of infertility stigma on infertile women and monitor them as early as possible to assess the damage done to them and identify effective responses to minimize the impact. Providers can render narrative group counseling, cognitive therapy, couple counseling, psychological interventions, and other measures to regulate patients' negative emotions. Simultaneously, caregivers should improve patients' health education, correct their misconceptions, and assist them in coping with negative experiences to lessen the impact of stigma on them.

## 9. Accessible summary

### 9.1. What is known on the subject?

- Due to various sociocultural factors and other types of influence, infertile women generally carry with them a sense of stigma, and that stigma itself brings great psychological burden to them and affect the treatment process.

### 9.2. What does this paper added to existing knowledge?

- Women with infertility are more likely to experience stigma than men. This study is the first systematic survey of its kind on the stigma in infertile women to explore in depth the impact of stigma on mental health and their quality of life.

### 9.3. What are the implications for practice?

- The results have found that the infertility stigma can bring heavy mental pressure and psychological burden to infertility patients and affect their quality of life.
- This study entails medical staff to pay attention to the mental health of infertility patients, so that they can develop more targeted and effective nursing intervention measures, to reduce the stigma of infertility patients, bring down their reproductive pressure, and improve their quality of life.

## Author contributions

YX: conceptualization, methodology, formal analysis, and writing—original draft. PY, LL, and YR: investigation. CN: resources. YZ, CN, and YX: writing—review and editing. All authors contributed to the article and approved the submitted version.
