# Supplementary material for: The impact of stigma on mental health and quality of life of infertile women: A systematic review
Source: Front Psychol. 2023 Jan 9;13:1093459. doi: 10.3389/fpsyg.2022.1093459 (PMC9869765; doi:10.3389/fpsyg.2022.1093459)
Supplement: Supplementary file 1 [file Table_1.DOCX]

| **Section and Topic** | **Item #** | **Checklist item** | **Location where item is reported** |
| --- | --- | --- | --- |
| **TITLE** | | |  |
| Title | 1 | The impact of stigma on mental health and quality of life of infertile women: a systematic review. | Title Page1，line10-13 |
| **ABSTRACT** | | |  |
| Abstract | 2 | Background: Stigma affects approximately 53.08%~64% of female infertility patients worldwide. Stigma not only causes harm to the mental health of patients, but also affects their quality of life, making them bear the adverse social consequences like domestic violence, marriage breakdown, or even delay of the treatment. Therefore, it is crucial to have a deep understanding and effective intervention in patients' stigma. Methods: The literature search used four English databases (Cochrane Library, EMBASE, Web of Science, and PubMed) and two Chinese databases (CNKI and Wanfang). The search time of the literature is from the establishment of the library to 2022, with no language restriction. The study was conducted under the guidance of the preferred reporting item for systematic reviews and meta-analyses (PRISMA) (Page et al., 2020). The Strengthening the Reporting of Observational Studies in Epidemiology guidelines (STROBE) were used to assess the quality of articles. The quality of all qualitative studies was assessed using Evidence-Based Care Guidelines tool. This study is registered on PROSPERO, number CRD42022357058. Results: The review included 30 studies, with 22 cross-sectional studies and 8 qualitative studies. This study found that social support, living environment, education level, occupation, and fertility awareness were the influencing factors of infertility stigma. Seventeen quantitative studies in this review looked at the effects of stigma on the mental health of women with infertility, which can cause psychological distress in patients with anxiety, depression, hopelessness, and grief. Five studies found that stigma had a negative impact on the quality of life of people with infertility, and when stigma increased, their quality of life decreased. Of the qualitative studies included in this review, eight found that stigma negatively affected women's quality of life and mental health. Conclusions: Infertility stigma can bring heavy mental pressure and psychological burden to infertility patients and affect their quality of life. Therefore, effective and targeted psychological interventions should be developed to reduce patients' stigma and improve their quality of life. To ensure the physical and mental health of infertility patients, healthcare workers must develop targeted nursing interventions, provide professional counseling services to reduce the level of stigma in patients, reduce fertility stress, and improve their quality of life. Funding: National Natural Science Foundation of China, Natural Science Foundation of Jiangsu Province, China, Natural science fund for colleges and universities in Jiangsu Province, China, Postgraduate Research & Practice Innovation Program of Jiangsu Province, China. | Abstract  Page1，line14-54 |
| **INTRODUCTION** | | |  |
| Rationale | 3 | We found that infertility stigma can bring heavy mental pressure and psychological burden to infertility patients and affect their quality of life. Although in recent years, scholars have conducted a series of studies on the impact of stigma on the mental health and quality of life of infertile women, there is no review to systematically summarize it. Therefore,this review aims to investigate whether disease stigma affects the mental health and quality of life of women experiencing fertility difficulties and provide a reasonable way for medical staff to develop effective interventions in the future, reduce patient stigma, reduce patient psychological burden, and improve quality of life. | INTRODUCTION  Page2，line13-40 |
| Objectives | 4 | The objective of this study was to use a descriptive systematic review to explore and summarize the stigma of female infertility patients and its impact on patients, and to provide a theoretical basis for the clinical treatment and nursing intervention of female infertility patients. | INTRODUCTION  Page3，line41-47 |
| **METHODS** | | |  |
| Eligibility criteria | 5 | The eligible studies had to meet all of the following criteria: (a) These studies must have been published, and the research methodology should be a cross-sectional study associated with female infertility stigma; (b) the subjects were infertile women; (d) the outcome measures included the association between stigma and mental health, or stigma and quality of life. (c) There is no restriction on the language and year of publication of the literature. We excluded studies of the following criteria: (a) Literature that does not match the content of this article. (b) The literature data is incomplete, and complete information cannot be obtained after contacting the author. (c) Meta, systematic reviews, and reviews of the literature. (d) The quality evaluation is low in the literature. We only look at the impact of stigma on the mental health and quality of life of women with infertility, not on the situation of men with infertility. We did not limit the study site or sample size. See Table 2 for details. We included mental health and quality of life as the outcome variables of this study. | Methods  Page 5，line12-25 |
| Information sources | 6 | We performed searches in the following databases:  o MEDLINE  o Embase (Excerpta Medica dataBASE)  o Cochrane Library  o Web of Science  o PubMed  o Wan-fang data  o CNKI  In addition, relevant citations from the included studies were searched by hand. | Methods  Page4，line21-34 |
| Search strategy | 7 | From the establishment of each database until July 2022, We conducted a comprehensive literature search without language restrictions using the databases of the Cochrane Library, EMBASE, Web of Science, PubMed, Wan-fang data and CNKI. Computer searches used Medical Subject Heading and keywords “infertility”, “subfertility”, “barreness”, “sterility”, “reproductive sterility”, “stigma”, “social stigma”, “perceived stigma”,“shame”, “discriminate”, “psychological”, “psychological stress”, “mental health”, “quality of life ”. Preliminary screening is carried out by thesis title and abstract. In addition, relevant citations from the included studies were searched by hand.  The following is the search query performed in the embase database:  #1 'infertility':ab,ti or 'subfertility':ab,ti or 'barreness':ab,ti or 'sterility':ab,ti or 'reproductive sterility':ab,ti  #2 'stigma':ab,ti or 'social stigma':ab,ti or 'perceived stigma':ab,ti or 'shame':ab,ti or 'discriminate':ab,ti  #3 'psychological':ab,ti or 'psychological stress':ab,ti or 'mental health':ab,ti or 'quality of life':ab,ti  #4 #1 and #2 and #3 | Methods  Page4，line21-34 |
| Selection process | 8 | In this study, two researchers completed the screening of information in the literature. After excluding the inconsistent literature, they continued reading the rest of the literature to determine if it could be included. The above tasks were done by two people independently. Finally, two researchers cross-checked the results, and if there was any disagreement during the period, a third party could be involved to discuss and resolve the disagreement. Missing or uncertain literature could be obtained by contacting the author by email. | Methods  Page5，line30-40 |
| Data collection process | 9 | We extract specific features of interest from each document, such as publication year, research methodology, data collection techniques, direction of research results, etc., and fill them into a designed table for analysis. When information regarding any of the above was unclear, we contacted authors of the reports to provide further details. | Methods  Page5，line30-40 |
| Data items | 10a | We describe important conclusions for each of the included literature in the results section of the table:  • Infertile women perceive a higher sense of stigma compared to men.  • Infertile women will have a sense of shame because of discrimination from the outside world, resulting in depression.  • Higher levels of perceived stigma were associated with increased infertility-related stress.  • Stigma can predict anxiety, depression, and psychological distress in patients.  • The stigma can cause patients to have social avoidance tendencies and distress in actual interactions  • The stigma of infertility can cause patients to develop negative emotions, such as anxiety and depression.  • Quality of life.  We extracted outcome indicators that assess the effect of stigma on a patient's psychological status and quality of life in some of the following terms:  • anxiety;  • depression;  • hopelessness;  • quality of life.  If several outcome measures appeared in a single document at the same time, they were all included. At the end, we extracted the adverse effects of stigma on mental health and quality of life.  If more than one scale related to psychology and quality of life is included in the literature, we include and extract data from it:  • Social Communication Questionnaire (SCQ),  • Fertility Problem Inventory (FPI),  • Beck Anxiety Inventory (BAI),  • Hospital Anxiety and Depression Scale (HADS),  • Experience of Shame Scale (ESS),  • Fertility quality of life (FertiQoL);  • any other scale. | Methods  Page13-19，line14-16 |
|  | 10b | We collected data on:  • the report: author, year, and source of publication;  • Study or cohort name, location, and commencement date  • Study design  • Funding sources and funder involvement in the study | Methods  Page13-19，line14-16 |
| Study risk of bias assessment | 11 | This review is a descriptive systematic review that summarizes the findings of the selected studies by grouping, clustering, proposing frameworks, feature lists, etc., and declaratively integrates accumulated evidence to draw conclusions and/or make recommendations, so no risk of bias assessment is conducted. | Methods  Page 5，line45-53 |
| Effect measures | 12 | Based on the information obtained from the included studies, we assessed the impact of stigma on mental health and quality of life in infertile patients through a tabular summary. The results section of the table describes the key conclusions of each included study. | n/a |
| Synthesis methods | 13a | The studies included in this systematic review are quantitative and qualitative, and the quantitative studies are cross-sectional observational studies, and the important results of each study are integrated using tabular descriptions. | n/a |
|  | 13b | By combining information from different research backgrounds, all aspects of the research problem are explored, and the contributions of each original research are described in turn, and the contradictory parts are explained. | n/a |
|  | 13c | We compiled some tables using Word documents. These tables include the source of the study, the number of participants, and a summary of the study for each major outcome. We summarize the impact of stigma on mental health and quality of life in infertile women. | n/a |
|  | 13d | This descriptive systematic review summarized the key findings in the article using a tabular summary. | n/a |
|  | 13e | The studies included in this systematic review were cross-sectional observational studies and qualitative studies, the results were summarized in tabular manner, and no heterogeneity was performed. | n/a |
|  | 13f | The studies included in this systematic review were cross-sectional observational studies and qualitative studies, the results were summarized in tabular manner, and no sensitivity analysis was performed. | n/a |
| Reporting bias assessment | 14 | To assess selective reporting bias, we compared the results hypothesized by the original investigator during the study period with the reports in the published papers by comparing the information section in the final publication. If there are no published study protocols, and the authors do not provide unpublished protocols as requested, we compared the methodological and results sections of published papers. We also use our knowledge of the clinical field to identify places where researchers are not reporting commonly used outcome measurements. | n/a |
| Certainty assessment | 15 | Two people (Yue Xie, Yue Ren) independently assessed the certainty of the evidence. The quality of articles was evaluated independently by these reviewers using Strengthening the Reporting of Observational Studies in Epidemiology guidelines (STROBE) (University of Bern, 2009). | Methods  Page 5，line45-53 |
| **RESULTS** | | |  |
| Study selection | 16a | We found 1,323 records in databases searching. After duplicates removal, we screened 1,153 records, from which we reviewed 387 full-text documents, and finally included 30 papers. Later, we searched documents that cited any of the initially included studies as well as the references of the initially included studies. However, no extra articles that fulfilled inclusion criteria were found in these searches (Fig 1). | RESULTS  Page8，line25-50 |
|  | 16b | Of the remaining 387 articles, 367 were excluded for a variety of other reasons (Fig. 1). Ultimately, this review included a total of 30 studies. All included studies were present in the initial database search. | RESULTS  Page 5，line45-53 |
| Study characteristics | 17 | The review included 30 studies, with 22 cross-sectional studies and 8 qualitative studies. Details are shown in Tables 2, 3, 4. | RESULTS  Page 5，line45-53 |
| Risk of bias in studies | 18 | This article is a descriptive systematic review, mainly through the collection, specification, generation and analysis of the included literature to integrate, using the STROBE assessment tool to assess the quality of the literature, without risk of bias assessment. | RESULTS  Page5-7，line45-36 |
| Results of individual studies | 19 | The results of the impact of stigma on mental health and quality of life in infertile women were summarized using the table. Details are shown in Tables 2, 3, 4. | RESULTS  Page13-19，line14-16 |
| Results of syntheses | 20a | For each synthesis, briefly summarise the characteristics and risk of bias among contributing studies. | n/a |
|  | 20b | This systematic review uses tables to summarize the results of the impact of stigma on mental health and quality of life in infertile women. | n/a |
|  | 20c | The studies included in this review were cross-sectional and qualitative, with no heterogeneous manifestations. | n/a |
|  | 20d | The studies included in this systematic review were cross-sectional and qualitative studies, and no sensitivity analyses were conducted. | n/a |
| Reporting biases | 21 | The studies included in this systematic review were cross-sectional and qualitative and did not report bias. | n/a |
| Certainty of evidence | 22 | Two investigators (Yue Xie, Yue Ren) assessed the certainty (or confidence) of the body of evidence for each outcome assessed. | RESULTS  Page5，line45-36 |
| **DISCUSSION** | | |  |
| Discussion | 23a | Prior to this systematic review, there was no systematic review of the mental health and quality of life of infertile women, and this study summarized the current research status of the stigma of infertile women and the impact on psychology and quality of life by combining 22 cross-sectional studies and 8 qualitative studies, and found that infertility-related stigma will have a negative impact on the psychology and daily life of infertile women. | Discussion  Page7-20，line43-24 |
|  | 23b | The research included in this systematic review is mainly cross-sectional studies and qualitative studies, and the decisive feature of such systematic reviews is that the textual orientation is adopted in the process of analysis and synthesis, so subjective bias will inevitably occur. | Discussion  Page22，line27-46 |
|  | 23c | Although the criteria and literature quality have been strictly controlled, this paper has some limitations. First, the findings might get impacted because each study had a different purpose and was measured using different research tools. Second, the included studies were predominantly cross-sectional with mixed literature quality, which may impact findings. Third, this paper only includes Chinese and English literature and lacks an evaluation of non-Chinese and non-English literature. | Discussion  Page22，line27-46 |
|  | 23d | This review found that stigma can adversely affect infertile women’s psychology and quality of life. Health workers and mental health professionals should be aware of the negative impact of infertility stigma on infertile women and monitor them as early as possible to assess the damage done to them and identify effective responses to minimize the impact. Providers can provide narrative group counseling, cognitive therapy, couple counseling, psychological interventions, and other measures to regulate patients’ negative emotions. Simultaneously, caregivers should improve patients’ health education, correct their misconceptions, and assist them in coping with negative experiences to lessen the impact of stigma on them. | Discussion  Page23，line15-31 |
| **OTHER INFORMATION** | | |  |
| Registration and protocol | 24a | Data sharing not applicable to this article as no datasets were generated or analysed during the current study. | n/a |
|  | 24b | The review protocol was registered with the International Prospective Register of Systematic Reviews (PROSPERO) database (registration number: CRD42022357058). | n/a |
|  | 24c | No revisions were made to the information provided at the time of registration or in the protocol. | n/a |
| Support | 25 | National Natural Science Foundation of China, Natural Science Foundation of Jiangsu Province, China, Natural science fund for colleges and universities in Jiangsu Province, China sponsored by Changmin Niu. Postgraduate Research & Practice Innovation Program of Jiangsu Province, China sponsored by Yue Xie. | Page24，line18-37 |
| Competing interests | 26 | The authors declare no conflicts of interest. | Page23，line49-51 |
| Availability of data, code and other materials | 27 | Data sharing not applicable to this article as no datasets were generated or analysed during the current study. | n/a |

*From:*  Page MJ, McKenzie JE, Bossuyt PM, Boutron I, Hoffmann TC, Mulrow CD, et al. The PRISMA 2020 statement: an updated guideline for reporting systematic reviews. BMJ 2021;372:n71. doi: 10.1136/bmj.n71

For more information, visit: <http://www.prisma-statement.org/>
